# Mixed Metal–Organic Framework Mixed-Matrix Membranes: Insights into Simultaneous Moisture-Triggered and Catalytic Delivery of Nitric Oxide using Cryo-scanning Electron Microscopy

**DOI:** 10.1021/acsami.3c11283

**Published:** 2023-10-11

**Authors:** Romy Ettlinger, Simon M. Vornholt, Madeline C. Roach, Robert R. Tuttle, Jonathan Thai, Maadhav Kothari, Markus Boese, Andy Holwell, Morven J. Duncan, Melissa Reynolds, Russell E. Morris

**Affiliations:** ‡ School of Chemistry, University of St. Andrews, North Haugh, St Andrews KY16 9ST, United Kingdom; ⊥ Department of Chemistry, Colorado State University, 1872 Campus Delivery, Fort Collins, Colorado 80523, United States; || ZEISS Research Microscopy Solutions, Carl-Zeiss-Straße 22, Oberkochen 73447, Germany; # Carl Zeiss Microscopy Ltd, Cambourne, Cambridge CB23 6DW, United Kingdom

**Keywords:** metal−organic frameworks, mixed-matrix membranes, composite materials, nitric oxide, medical applications, Cryo-SEM

## Abstract

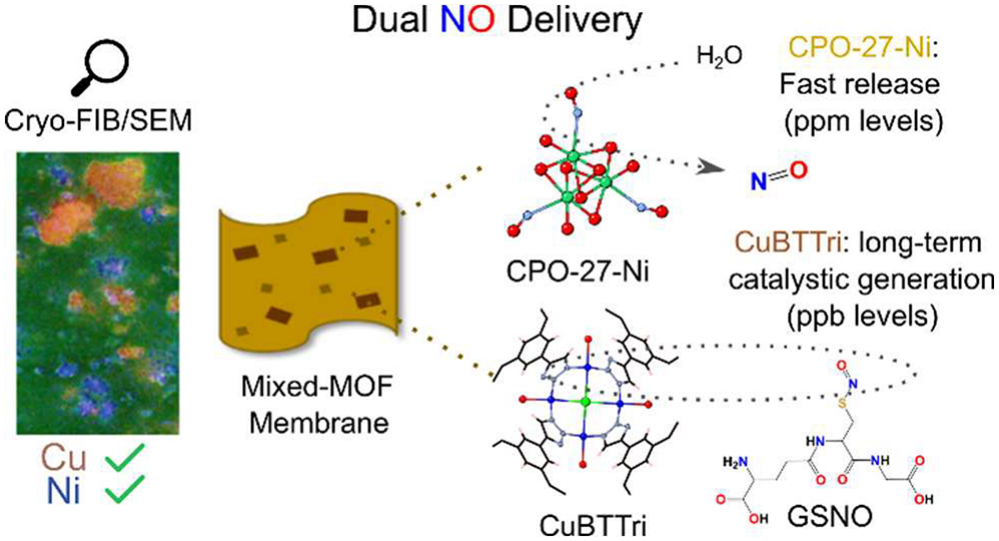

The fundamental chemical and structural diversity of metal–organic frameworks (MOFs) is vast, but there is a lack of industrial adoption of these extremely versatile compounds. To bridge the gap between basic research and industry, MOF powders must be formulated into more application-relevant shapes and/or composites. Successful incorporation of varying ratios of two different MOFs, CPO-27-Ni and CuBTTri, in a thin polymer film represents an important step toward the development of mixed MOF mixed-matrix membranes. To gain insight into the distribution of the two different MOFs in the polymer, we report their investigation by Cryo-scanning electron microscopy (Cryo-SEM) tomography, which minimizes surface charging and electron beam-induced damage. Because the MOFs are based on two different metal ions, Ni and Cu, the elemental maps of the MOF composite cross sections clearly identify the size and location of each MOF in the reconstructed 3D model. The tomography run was about six times faster than conventional focused ion beam (FIB)-SEM and the first insights to image segmentation combined with machine learning could be achieved. To verify that the MOF composites combined the benefits of rapid moisture-triggered release of nitric oxide (NO) from CPO-27-Ni with the continuous catalytic generation of NO from CuBTTri, we characterized their ability to deliver NO individually and simultaneously. These MOF composites show great promise to achieve optimal dual NO delivery in real-world medical applications.

## Introduction

To pave the way for the transition of highly porous and versatile metal–organic frameworks (MOFs) from the benchtop toward real-world applications, it is important to improve their industrial processability.^1,2^ One commonly applied strategy is to pelletize MOF powders, but this attenuates their properties due to the binder required and may also change their structural properties due to the applied pressure.^3^ An alternative approach is to incorporate the active material into stand-alone polymer films by producing MOF composites, also often referred to as mixed-matrix membranes (MMMs).^4−8^ For this approach, it is important to make sure that the porous interior of the MOF is still accessible, as this preserves its application-relevant properties (e.g., accessibility of internal porosity and open metal sites).^9^ So far MOF-based MMMs have proven particularly useful for the separation of gases or molecules,^10−13^ sensing,^14−16^ light-harvesting applications,^15^ and biomedical applications.^17−19^ Studying the interface between the MOF filler and polymer is challenging and often requires significant effort and resource. However, some groups have characterized the interface using computational studies paired with spectroscopic methods.^8,20,21^

Recently, we were able to shed light on a selected MOF composite system where we successfully elucidated the interactions between the MOF CPO-27-Ni and its surrounding polymer polyurethane (PU).^18^ Advanced focused-ion-beam scanning electron microscopy (FIB-SEM) paired with computational analyses revealed that the microstructures of the CPO-27-Ni framework were preserved in the polymer network.^18^ The dispersion of the MOF at various wt % loadings was studied, and it was concluded that the amount of active material had a profound effect on the targeted application: the delivery of gaseous nitric oxide (NO). Overall, composites with 10 wt % MOF proved to be best performing.^18^ The MOF CPO-27-Ni has been identified as a promising NO storage/release agent as its coordinatively unsaturated sites allow large quantities of gas to be chemisorbed and for near total release within hours when triggered by moisture. This unique ability to deliver the gaseous NO, which is a well-known antimicrobial agent, in a controlled manner makes such MOF composites interesting materials for applications in medical devices that could locally prevent healthcare associated infections.^22^

While one mechanism of NO delivery involves coordination to unsaturated sites in the MOF CPO-27-Ni,^23,24^ we have also shown that another mechanism can be employed. Specifically, the catalytic production of NO by the MOF CuBTTri is by oxidation of GSNO (S-nitrosoglutathione), which is a tripeptide that originates from within the human body. In our previous work we showed that GSNO can act as an endogenous source of NO in the presence of CuBTTri.^9^ In this NO delivery application, the NO is produced in smaller doses but over a much longer time (potentially indefinitely), which may create a permanent antimicrobial surface to prevent medical device fouling. This combination approach would be very useful by providing a burst of NO to provide an initial kill of surface bacteria, followed by steady NO production that prevents further colonization and infection.

In this work, we successfully combined these two different NO delivery mechanisms in one material by embedding a combined total loading of 10 wt % of varying ratios of two MOFs, CPO-27-Ni and CuBTTri, in a (medical grade) PU matrix.

To gain deeper insight into the distribution and microstructure of the two MOFs in the novel mixed MOF composites, focused ion beam scanning electron microscopy (FIB-SEM) was utilized. This powerful tool provides three-dimensional (3D) information on the microstructures embedded into a support matrix.^7,25^ Studying the interface on classical SEMs is of limited use since polymer matrices typically suffer from charging and/or the electron beam can alter or even damage the embedded MOFs. Therefore, this work highlights the benefits of using an advanced cryogenic FIB-SEM (Cryo-FIB-SEM) technique. Owing to the significantly increased stability of the MOF composites on the cryo-stage, which are otherwise difficult to analyze, first insights into image segmentation could be successfully combined with machine learning. The Cryo-FIB-SEM technique also allowed for the analysis of several cross sections of the MOF composite films and enabled 3D reconstruction of the microstructures of the MOFs and unequivocal identification of their position in the polymer membrane.

To validate the ability of the two different MOF structures to deliver NO in medical applications, independent of their embedding in a polymer matrix, the potential for dual NO delivery was evaluated utilizing the respective methods individually and simultaneously: the adsorption and short-lived, burst release of gaseous NO by CPO-27-Ni when moisture is present (with relatively high, parts per million levels of NO) and the constant long-lived catalytic production of NO by CuBTTri (with lower, parts per billion levels of NO generated).

## Results and Discussion

### Material Preparation and Characterization

**Figure 1:**
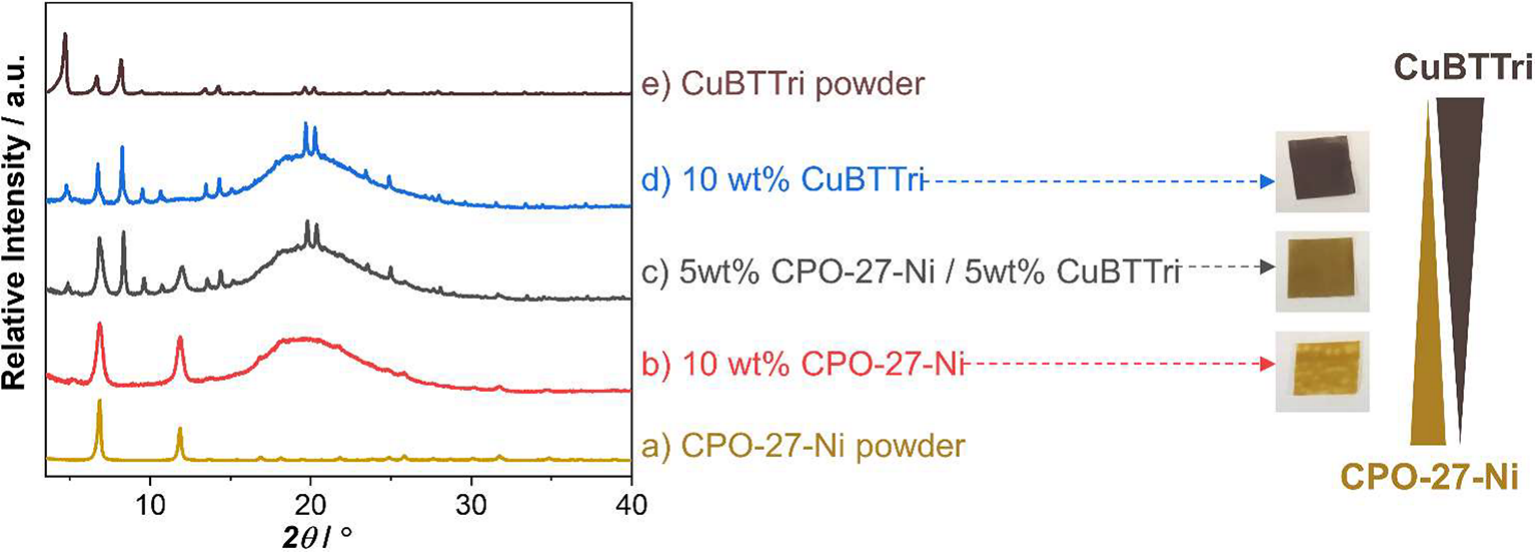
X-ray powder diffraction patterns of (a) CPO-27-Ni and (e) CuBTTri powder, and their MOF/polymer composites containing different ratios of CPO-27-Ni to CuBTTri, namely (b) 10 wt %:0 wt %, (c) 5 wt %:5 wt %, and (d) 0 wt %:10 wt % (left), and photographs of the respective MOF composite films (right).

Composites containing the two MOFs CPO-27-Ni and/or CuBTTri were prepared by synthesizing the respective MOF powders based on methods previously described in the literature.^17,18^ A total MOF loading of 10 wt % (with respect to the mass of the polymer) was chosen as the active material, as our previous studies found that at this loading level the gas transport properties for NO through the polymer matrix was optimal.^18^ Different ratios of CPO-27-Ni to CuBTTri of 100:0, 50:50, and 0:100 were embedded, which correspond to 10 wt % CPO-27-Ni, 5 wt % CPO-27-Ni and 5 wt % CuBTTri, and 10 wt % CuBTTri, respectively. The desired quantities of MOF powders were mechanically dispersed in ethanol, added to the viscous PU polymer slurry, and the MOF/polymer slurry was then cast as a thin film using the doctor blade method, leaving free-standing MOF composite films (Figure 1).

Photographs of the MOF composite films are shown in Figure 1 and show a uniform color, suggesting good dispersion of the MOF particles in the otherwise colorless polymer. The films show a continuous color change from a caramel tone at 10 wt % CPO-27-Ni to a dark brown at 10 wt % CuBTTri (Figure 1). The films are smooth to the touch and have similar thickness (∼100–105 μm), further implying that the MOF particles are well embedded within the polymer matrix. The powder X-ray diffraction patterns of the resulting MOF composite films confirm the presence of the two MOFs (Figure 1 and Figure S1). The broad amorphous region (15–25° 2θ) is attributed to the polymeric matrix. EDX analysis proved useful to determine the elemental ratios of Ni:Cu in the composites with a 50:50 ratio of CPO-27-Ni to CuBTTri. The measured metal ratio of 49.3:50.7 ± 4.3 was in good agreement with the expected 50:50 ratio.

**Figure 2:**
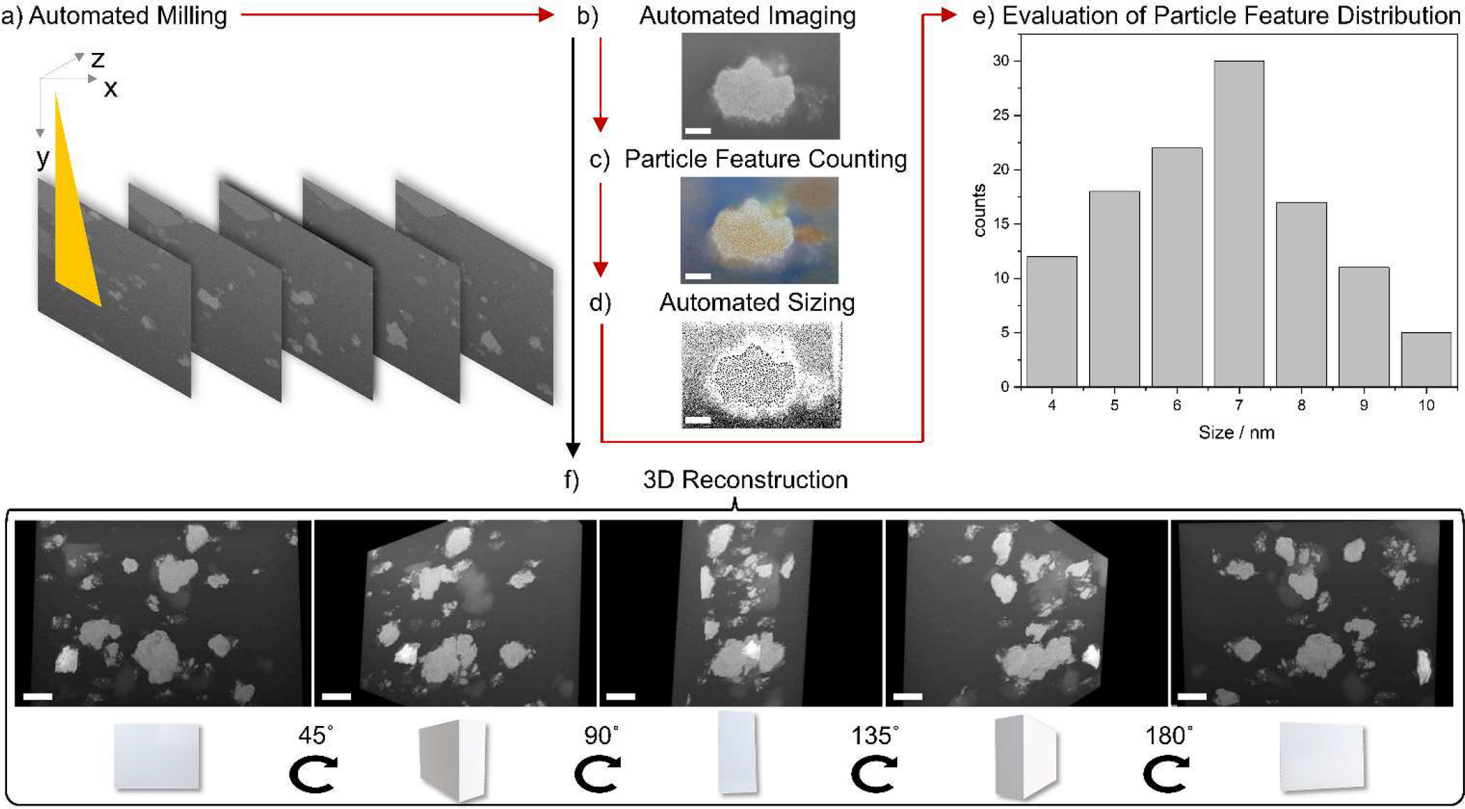
Overview of the automated SEM analysis using a ZEISS Crossbeam 550L with a Quorum Cryo-stage: (a) automated milling; (b) automated imaging; (c) particle feature counting; (d) automated sizing; (e) evaluation of particle feature distribution; and (f) 3D reconstruction of a MOF composite with a 50:50 ratio of CPO-27-Ni and CuBTTri; scale bar: (b–d) 200 nm and (f) 500 nm.

To further analyze the size and distribution of the MOF particles in the composite, electron microscopy in combination with FIB-milling was carried out (Figure 2). As these experiments require a FIB-SEM that generates slices that are separately imaged (Figure 2a), these experiments are often carried out over multiple hours to generate a reasonable volume of interest, even for reconstructed micrographs of only ∼10 μm^3^. Sample drift is especially problematic for such 3D reconstructions as it significantly increases the difficulty for automated programs during the lengthy image acquisition (autofocus, autocontrast).

To demonstrate the outstanding resolution of micrographs and accompanying EDS maps arising from imaging using the Cryo-FIB-SEM, the same MOF composites were also imaged using two standard SEMs, namely, a JSM-IT800 Schottky Field Emission SEM (IT-800) and a FEI Scios Dual Beam which is equipped with a FIB column (Scios) (details see Supporting Information Table S1). While the micrographs recorded with the standard SEMs revealed common drawbacks such as profound charging on the surface (Table S1a and d) and severe “curtaining” (Table S1b and e), these issues were overcome using the ZEISS Crossbeam 550L with a Quorum Cryo-stage without gold coating the sample. Instead, a different preparation of the MOF composite film is involved: first, a plunge freeze method to prevent ice crystal buildup, followed by an air-free transfer to afford a contaminant- and condensation-free sample. As shown in Figure 2 and Table S1c, f using this advanced Cryo-FIB-SEM technique allowed us to obtain a clear cross section of the MOF composite. Compared to the images obtained through other instruments, the resulting micrographs show the embedded MOF particles in the polymer membrane in greater detail and even reveal substructures in individual MOF particles. Owing to this good resolution (∼3 nm), first insights into image segmentation combined with machine learning could be achieved. Subsequent to the automated milling, automated imaging, particle feature counting, and sizing could be applied (Figure 2b–e). Evaluating the particle feature size distribution within one particle of the full 3D stack of 10 nm slices in a progressive slice by slice calculation gave a particle feature range of 4–10 nm with a maximum at 7 ± 3 nm. Such precise, autonomous material milling in combination with automatic image evaluation is of great interest for efficient high-throughput analysis of samples. The Cryo-FIB-SEM assisted milling and subsequent 3D reconstruction revealed that the microstructure of the MOF is well dispersed and nonaggregated throughout the entire depth of the film. Previous research has identified that this dispersion and separation of MOF particles within the support matrix is required for optimal gas transport.^18^

To further illustrate the utility of Cryo-FIB-SEM, a 3D reconstruction of the MOF composite was performed with a film containing a 50:50 ratio of CPO-27-Ni to CuBTTri (Figure 2f). In this study, a volume as large as 16 μm × 6 μm × 10 μm (∼960 μm^3^) of the MOF composite was FIB processed. To maximize the resolution of the 3D reconstruction, a large number of cross-sectional cuts were made using a precise step width of 2 nm. The tomography run of ∼960 μm^3^ was complete in 1.5 h (about 6× faster than conventional FIB-SEM) and the 3D data were reconstructed within a few minutes (Figure 2f and Video S1).

**Figure 3:**
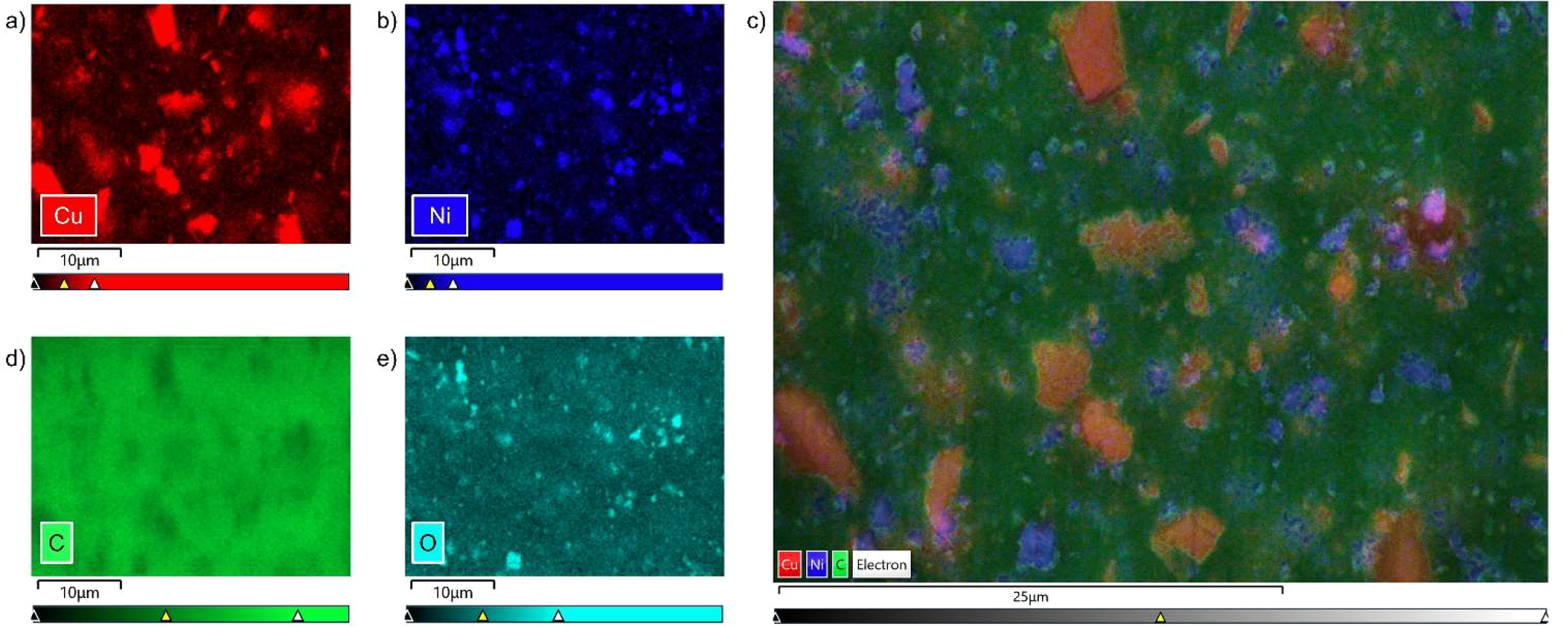
EDS elemental mapping of MOF composite with a 50:50 ratio of CPO-27-Ni and CuBTTri: the compositional maps of (a) Cu (red), (b) Ni (dark blue), (d) C (green), (e) O (light blue), and (c) an overlay of the compositional maps of Cu (red), Ni (dark blue), and C (green).

To visualize the position of the two different MOFs (CPO-27-Ni and CuBTTri) in the composite, EDS elemental maps were collected by using the unique metals of Ni and Cu, respectively. Unlike conventional EDS whereby a sample is coated with gold to increase conductivity to generate a higher quality image, Cryo-FIB-SEM does not require the sample to be coated, thereby eliminating any interference from this additional gold signal during data collection. For each cross section of the MOF composite films, the sample was milled with a FIB under cryogenic conditions yielding a clean surface, and an accurate EDS scan was recorded at room temperature to facilitate a faster count rate of these dispersed electrons. Figure 3 shows the results of the EDS analysis of the MOF composite with a 50:50 ratio of CPO-27-Ni and CuBTTri. Compositional maps were recorded for the elements Cu, Ni, C and O. The elements Cu and Ni, which are only part of either CuBTTri or CPO-27-Ni, respectively, explicitly identify the position of the two different MOF particles in the polymer. The overlay of the recorded compositional map of Cu (Figure 3a) and Ni (Figure 3b) in Figure 3c shows that the two MOFs do *not* significantly overlap but are distributed homogeneously throughout the entire depth of the polymer matrix. These micrographs can be used to determine the particle sizes of the two different MOFs. The calculated particle sizes are 3.45 ± 1.21 μm for CuBTTri and 1.12 ± 0.45 μm for CPO-27-Ni, which is in good agreement with the values obtained from PSD analysis of MOF dispersions after ball-milling in EtOH/THF (∼3.99 and ∼1.22 μm, respectively, Table S2).

The compositional map of carbon, which is part of the polymer and both MOF linkers, reveals that it is found throughout the whole cross section but shows slightly lower intensities (i.e., density) in the areas where the porous CuBTTri and CPO-27-Ni particles are found (Figure 3d). Oxygen was detected throughout the entire cross section with much higher intensity in the areas where CPO-27-Ni is present likely because O is a larger atomic fraction of the CPO-27 linker (C_8_H_6_O_6_) than that of the polymer (C_3_H_8_N_2_O) (Figure 3e).

### NO Loading and Delivery Behavior

We first recorded the NO release profiles of the MOF composite films containing either CPO-27-Ni or CuBTTri at 10 wt % loading in the medically relevant PU polymer. The two MOFs deliver NO through different mechanisms. NO binds to open metal sites in CPO-27-Ni after activation and then NO released at ppm levels is triggered by moisture.^18^ CuBTTri catalytically generates NO (at ppb levels) in the presence of GSNO, which is an endogenous tripeptide available within the bloodstream.^26^

In the first study, the moisture triggered release of preadsorbed NO by the MOF composites were recorded. For this purpose, the films were activated at 80 °C *in vacuo*, loaded with NO for 1 h, and data were acquired when exposed to 11% relative humidity (Figures S4 and S5 and Table S3). Traditionally, these MOF materials are activated above 100 °C *in vacuo*; however, the polyurethane polymer used to fabricate the composite material is thermoplastic with a softening point above 85 °C, therefore milder activation conditions are required for the composite material.^18^ The samples containing 10 and 5 wt % CPO-27-Ni showed a burst release of NO for a short duration, followed by a slower release of adsorbed NO over the course of 19.3 and 9.8 h before returning to the baseline, respectively (Figure S4). As expected, the MOF composite containing 0 wt % CPO-27-Ni with 10 wt % CuBTTri displayed negligible moisture-triggered release of NO over the course of 3.2 h, due to the lack of available open metal sites. As the amount of CPO-27-Ni present in the MOF composite decreases, the overall amount of NO released decreases from 1.2 mmol g^–1^ for the 10 wt % CPO-27-Ni sample to 0.54 mmol g^–1^ for the 50:50 MOF composite (i.e., 5 wt % CPO-27-Ni), while composite materials containing 10 wt % CuBTTri (i.e., 0 wt % CPO-27-Ni) released only 0.01 mmol g^–1^. The trend in NO release versus composite composition underlines that controlled, triggered released of preadsorbed NO (in ppm levels) can be fully attributed to CPO-27-Ni within the polymer matrix, and that the polymer itself plays no part in any triggered release. The high initial burst release of NO is predicted to kill the bacteria on the surface of the MOF composite.^18,19^

In a second study, the ability of CuBTTri to catalytically generate NO was evaluated. In these experiments, the MOF composites (not loaded with NO) were submerged in a PBS solution (pH 7.4), and then, the oxidation of GSNO was tracked through chemiluminescence-based NO detection (Figure S6). NO production from GSNO was observed in parts per billion levels over the course of 1 h from the MOF composites containing 10 and 5 wt % CuBTTri (Figure S6), while there is no sustained GSNO to NO conversion catalysis observed for the sample with 0 wt % CuBTTri (i.e., 10 wt % CPO-27-Ni) as there are no Cu sites available. To prove that it is the Cu sites that are catalytically oxidizing the GSNO, a source of Cu ions (CuCl_2_ solution) was added to the reaction cell containing the sample with 10 wt % CPO-27-Ni (Figure S6a), which at that point resulted in a large spike in the amount of NO released. The trend in NO generation versus CuBTTri content is consistent with the catalytic activity of CuBTTri for GSNO oxidation. The minor spike in NO generation above baseline observed just after the GSNO addition for the sample with 10 wt % CPO-27-Ni is attributed to minor spontaneous oxidation of GSNO under the experiment conditions. Therefore, we infer that CuBTTri in this sample is responsible for the observed NO generation. The data qualitatively show that the MOF composites containing 5 wt % CuBTTri and 5 wt % CPO-27-Ni retain the catalytic GSNO to NO conversion activity observed for the neat CuBTTri powder.

**Figure 4:**
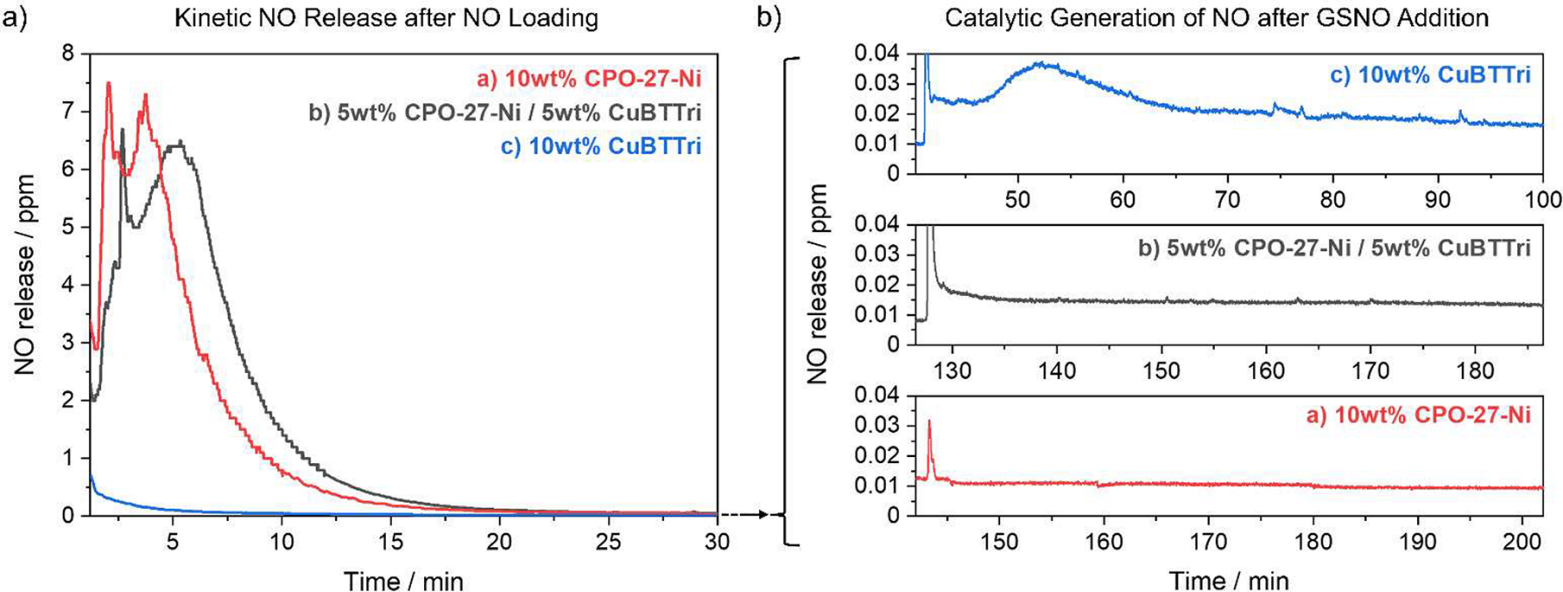
Dual NO delivery of MOF composite with a ratio of CPO-27-Ni to CuBTTri of (a) 10 wt %:0 wt %, (b) 5 wt %:5 wt %, (c) 0 wt %:10 wt % over time: (a) kinetic NO release in PBS (pH 7.4, left); and (b) catalytic generation of NO after GSNO addition (right).

Finally, the two different NO delivery mechanisms were investigated *simultaneously* in a combined dual NO delivery study (Figure 4): (i) the MOF composites were first activated and then allowed to dwell in a bath of NO, and then their kinetic NO release was recorded when submerged in PBS (Figure 4a); and (ii) once the NO release level fell below ∼10 ppb, fresh PBS as well as GSNO were injected into the reaction cell to track the catalytic generation of NO (Figure 4b). Similar to the first study of the kinetic NO release, the samples with 10 and 5 wt % CPO-27-Ni show a burst release of NO. As the samples were fully submerged in PBS, i.e., 100%RH, the moisture-triggered release occurred over 2.4 and 2.1 h, respectively, at a faster rate than when exposed to 11% relative humidity. The samples containing 0 wt % CPO-27-Ni with 10 wt % CuBTTri show almost no release of NO over the course of 0.7 h. Subsequently, fresh PBS and GSNO were added to elucidate whether the composite retained the ability to produce NO by oxidizing GSNO (Figure 4b). The addition of fresh PBS caused a spike for all samples, which can be attributed to the rapid expulsion of headspace volume (and hence trapped NO gas) in the reaction cell. For the sample containing 10 wt % CPO-27-Ni, the addition of GSNO did not result in sustained NO production and the NO release level continued to decrease. After addition of fresh PBS and GSNO, the samples containing 10 and 5 wt % CuBTTri catalytically generated NO in ppb levels above the baseline.

## Conclusion

In this study, we have reported the embedding of varying ratios of two different MOFs, CPO-27-Ni and CuBTTri, into a polyurethane membrane and investigated the microstructure of these materials using Cryo-SEM. SEM micrographs with a resolution of 3 nm could be recorded. Respective EDS element maps collected during the milling process allowed the reconstruction of a 3D model, revealing the two well-dispersed MOF structures in a volume as large as 960 μm^3^. Additionally, these first insights into image segmentation combined with machine learning pave the way toward automated sample analysis. As a result, we propose that Cryo-SEM analyses result in faster (less than 2 h) and more precise (resolution of 3 nm) evaluation of difficult samples, such as different MOF structures in a polymer, versus traditional SEM.

The free-standing mixed MOF composites were also investigated for sequential NO delivery through two different mechanisms in the context of medical device applications. The prepared membranes exhibit ideal qualities for next-generation NO releasing materials in medical devices because: (i) they provide rapid NO release at ppm levels from CPO-27-Ni to deliver a first antibacterial NO effect, followed by (ii) long-term catalytic NO generation by CuBTTri at ppb levels, which may prevent long-term medical device fouling.

## Materials and Methods

All chemicals used were sourced from common suppliers without further purification.

### Synthesis of CPO-27-Ni Powder

CPO-27-Ni powders were synthesized according to literature procedures published elsewhere.^27^ The powder X-ray diffraction pattern of the resulting yellow-brown powder proved phase pure in comparison to the CIF standard [CCDC# 2060146 (Figure S1)].^28^

### Synthesis of H_3_BTTri and CuBTTri powder

The H_3_BTTri ligand and CuBTTri were synthesized based on methods previously described in the literature.^17,29^ The resulting CuBTTri product was dried under reduced pressure overnight, and a Soxhlet extraction of the as-synthesized CuBTTri powder was conducted for 3 days, using 150 mL of methanol per 1 g of MOF; fresh methanol was used every 24 h. The powder X-ray diffraction patterns were recorded of both the as-synthesized material and after the solvent exchange, and both diffraction patterns (Figure S1) show peaks corresponding to the CuBTTri structure as compared to structures reported in the literature.^30,31^

### Preparation of MOF Composites

For the preparation of 10 wt % MOF/polymer composites, different ratios of CPO-27-Ni to CuBTTri, namely, 10 wt % CPO-27-Ni, 5 wt % CPO-27-Ni and 5 wt % CuBTTri and 10 wt % of CuBTTri, were investigated. To optimize the particle size distribution (PSD) of CPO_27-Ni, the MOF powder was dispersed in different solvents (THF and EtOH), for varying length of time (1 h, 1.5 and 3 h) and at different frequencies (10 and 15 Hz) (Table S2 and Figure S2). Using EtOH and operating the dual jar shaker mill at 15 Hz for 3 h yielded the narrowest PSD centered at ∼1.22 μm for CPO-27-Ni and these conditions were also employed for CuBTTri, yielding a PSD of ∼3.99 μm (Table S2 and Figure S3). These optimized MOF dispersions were then added to a viscous polymer solution [medical grade PU (CAS number: 68084-39-9, Sigma-Aldrich) dissolved in THF] to obtain 10 wt % of MOF calculated per gram of polymer. The MOF composites were cast as thin films by using the doctor blade method, resulting in a free-standing MOF composite film (∼100–105 μm thickness) after evaporation of the solvent (Figure 1).

### Kinetic NO Loading and Release

Activation and NO loading were performed in a similar protocol as previously reported.^4^ In short, each MOF composite was cut into 2 × 2 cm squares and activated for 16 h (80 °C, 10^–3^ mbar). This thermal treatment removes occluded solvent molecules and creates open metal Ni-sites in the case of CPO-27-Ni. After activation, the samples were introduced to a NO atmosphere (2 bar absolute pressure) for 1 h, after which the remaining NO was replaced with argon; kinetic release experiments were performed within 1 day. For the release of NO from the MOF composites under a relative humidity of 11%, samples were placed into a release chamber, and the gaseous NO was recorded (NOA, 280i, Zysense, Weddington, North Carolina).^5^ Data acquisition was stopped once the release levels fell below ∼10 ppb. From the recorded amounts of NO a concentration profile is obtained, which can be mathematically transformed into a total release of NO per mass of MOF within the film. Each measurement was recorded in duplicate.

### Catalytic Generation of NO

Prior to the experiment, the free-standing MOF composites were *not* NO loaded to ensure that the MOFs themselves do not contain any surface NO. Three 1 cm diameter circular samples were cut from the MOF composite film. To track *S*-nitrosoglutathione (GSNO) oxidation, the Nitric Oxide Analyzer (NOA, 280i, Zysense, Weddington, North Carolina) utilizing chemiluminescence-based NO detection were used. Phosphate-buffered saline (PBS, pH = 7.4) was added to a custom reaction cell to a total volume in the cell of 3 mL. The 1 cm diameter circular samples were added to the cell and fully submerged in the PBS solution. After initiating data collection on the NOA, 10 μM GSNO was injected into the reaction cell. The reaction was shielded from light using aluminum foil and proceeded at room temperature (approximately 20 °C) for 1 h with a collection interval of 1 s. For the sample composite containing no CuBTTri, i.e., 10 wt % CPO-27-Ni, after 1 h of baseline level signal, 10 μL of 0.1 M CuCl_2_ was added to ensure that the GSNO present was capable of releasing NO, a positive control for the experiment. The resulting NO release was plotted against time.

### Dual NO Delivery

Both previously described NO delivery mechanisms were combined. The MOF composites were first cut into 1 × 1 cm squares and activated for 16 h (80 °C, 1 × 10^–3^ mbar). Then the samples were introduced to a NO atmosphere (2 bar absolute pressure) for 1 h, after which the remaining NO was replaced with argon. To record their initial kinetic NO release, the MOF composites were submerged in 3 mL of PBS (pH 7.4) and data acquisition with a collection interval of 1 s was started. Once the NO release level fell below ∼10 ppb, another 3 mL of PBS and 10 μM GSNO was injected into the reaction cell to track the catalytic generation of NO. The reaction was shielded from light using aluminum foil and proceeded at room temperature (approximately 20 °C) for another 1 h. Each measurement was recorded in duplicate and the resulting NO release was plotted against time.

### Structural Characterization

Powder X-ray diffraction (PXRD) patterns were recorded on a STOE STADI/P diffractometer using Cu Kα1 radiation at room temperature from 3 to 50° (2θ). Particle size distributions (PSDs) of dispersed MOF powders were measured using a Malvern Mastersizer 2000 light scattering particle size analyzer, employing ethanol (EtOH) and tetrahydrofuran (THF) as the dispersion media. Three measurements were taken under stirring (1800 rpm) and a further three were recorded (under stirring) after a 3 min sonication cycle; each set of three measurements were averaged. SEM micrographs were collected using three different electron microscopes. A JEOL JSM-IT800 microscope was used at a working distance of 4 mm and an operating voltage as low as 3 kV by placing the free-standing MOF composites on copper tape. A Scios DualBeam at a working distance of 7 mm and low operating voltages (2–5 kV) to ensure sensitive mapping of the surface. The free-standing MOF composites were placed on copper tape and gold coated using Quorum Q150R ES sputter coater (10 mA, 30 s) prior to recording. For Cryo-SEM analysis the Microscope ZEISS Crossbeam 550L with a Quorum Cryo-stage was used. FIB milling was performed using small 2 nm steps and imaging conditions of 2.00 kV at −150 °C, imaged using an InLens detector, FIB probe 30 kV:700pa in analytical mode. Subsequently, automated stitching of FIB-SEM slices using Atlas 5 3D software enabled creation of 3D models. Live imaging at high resolution during milling enables faster tomography runs. In this study, a 16 μm × 6 μm × 10 μm volume was FIB processed in a 1.5 h tomography run and the 3D data was reconstructed within a few minutes. NO release measurements were recorded on a Nitric oxide analyzer (NOA, 280i, Sievers) using chemiluminescence technique. The measurements for a kinetic release were run in duplicates and those for catalytic generation of NO in triplicates.

## Supporting Information Available

PXRD patterns, particle size distributions, SEM micrographs, and NO release profiles (PDF)
Video S1, tomography video (AVI)

## Supplementary Material

am3c11283_si_001.pdf

am3c11283_si_002.avi

## Notes

The research data underpinning this work can be accessed at doi.org/10.17630/fa3d7e31-ff9c-45ad-a5d1-db2d03f8011c.

## Abbreviations

Cryo-SEM: cryo-scanning electron microscopy
EtOH: ethanol
FIB-SEM: focused ion beam scanning electron microscopy
GSNO: S-nitrosoglutathione
MMM: mixed matrix membranes
MOF: metal–organic framework
NO: nitric oxide
PSD: particle size distribution
PU: polyurethane
RH: relative humidity
THF: tetrahydrofuran.
