# Supplementary material for: Mixed Metal–Organic Framework Mixed-Matrix Membranes: Insights into Simultaneous Moisture-Triggered and Catalytic Delivery of Nitric Oxide using Cryo-scanning Electron Microscopy
Source: ACS Appl Mater Interfaces. 2023 Oct 11;15(42):49835–42. doi: 10.1021/acsami.3c11283 (PMC10614190; doi:10.1021/acsami.3c11283)
Supplement: Supplementary file 1 — am3c11283_si_001.pdf [file am3c11283_si_001.pdf]

Supporting Information

Mixed Metal-Organic Framework Mixed-Matrix Membranes: Insights into Simultaneous Moisture-Triggered and Catalytic Delivery of Nitric Oxide using Cryo-Scanning Electron Microscopy

Romy Ettlinger,[‡1] Simon M. Vornholt,[‡1†] Madeline C. Roach,[2] Robert R. Tuttle,[2] Jonathan Thai, [2] Maadhav Kothari,[3] Markus Boese,[3] Andy Holwell,[4] Morven J. Duncan,[1] Melissa Reynolds,[2] and Russell E. Morris[1*]

AUTHOR ADDRESS

[1] School of Chemistry, University of St Andrews, North Haugh, KY16 9ST St Andrews, United Kingdom; Email: rem1@st-andrews.ac.uk

[2] Department of Chemistry, Colorado State University, 1872 Campus Delivery, Fort Collins, CO, 80523, US

[3] ZEISS Research Microscopy Solutions, Carl-Zeiss-Straße 22, 73447 Oberkochen, Germany

[4] Carl Zeiss Microscopy Ltd, Cambourne, Cambridge CB23 6DW, United Kingdom

Contents

1. Characterization of MOF Powders and MOF Composites 2

2. NO-Release Studies 7

1. Characterization of MOF Powders and MOF Composites

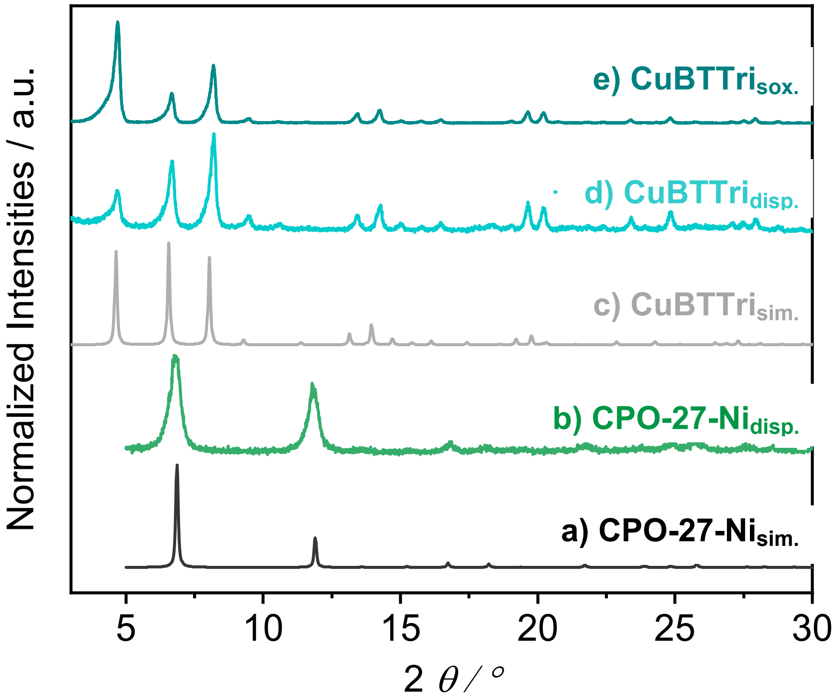

Figure S1: Comparison of X-ray diffraction powder patterns of a) CPO-27-Ni$_{sim.}$ (dark gray), b) CPO-27-Ni$_{disp}$ (green), c) CuBTTri$_{sim.}$ (light gray), d) CuBTTri$_{disp.}$ (light blue) and e) CuBTTri$_{sox.}$ (dark blue).

Comparison of three SEM Analyses

In the case of the standard SEM, a JSM-IT800 Schottky Field Emission SEM (IT-800), the MOF composite was analyzed without any further pretreatment to contrast functionality compared to Cryo-stage SEM (**Table S2a** and **d**). With these settings, the electron beam caused profound charging on the surface resulting in damage to the polymer film. Owing to the high sensitivity of the film to the beam, the sample drifted during image acquisition, resulting in low quality, blurred images, which are lacking in detail.

A step up from this classical SEM is the FEI Scios Dual Beam which is equipped with a FIB column (Scios). Despite the higher resolution given by this Dual Beam FIB-SEM, the composites still had to be coated with gold, to further improve conductivity (**Table S2b** and **e**). This gold coating further improves the sample stability and minimizes charging and sample drift. With these further steps, the materials' cross section could be investigated, but severe 'curtaining' appeared in the final image leaving an overall less defined and textured cross-section as the rather high energy of the FIB used for milling is not selective enough to mill away the soft and beam sensitive polymer with high accuracy. (**Table S2b**).

The best micrographs with a very clear cross section of the MOF composite were recorded using the 550L Zeiss Crossbeam with a Quorum Cryo-stage (**Table S2c** and **f**). FIB milling was performed using small 2 nm steps and imaging conditions of 2.00 kV at -150°C, imaged using an InLens detector, FIB probe 30kV:700pa in analytical mode. Subsequently, automated stitching of FIB-SEM slices using Atlas 5 3D software enabled creation of 3D models. Live imaging at high resolution during milling enables faster tomography runs. In this study, a 16 µm x 6 µm x 10 µm volume was FIB processed in a 1.5 h tomography run and the 3D data was reconstructed within a few minutes.

Table S1: Comparison of the micrograph quality of a film with a 50:50 ratio of CPO-27-Ni and CuBTTri originating from different SE microscopes with different analysis conditions, i.e. gold coating and/or cryogenic stage temperature of -150C: a,c) JSM-IT800, b.d) FEI Scios Dual Beam, and c) 550L Zeiss Crossbeam with a Quorum Cryo-stage; scale bar: a) 10 µm, b,c) 5 µm, and d-f) 2 µm.

	IT-800	Scios	Cryo Stage
Gold Coating	✗	✓	✗
Cryogenic Stage	✗	✗	✓

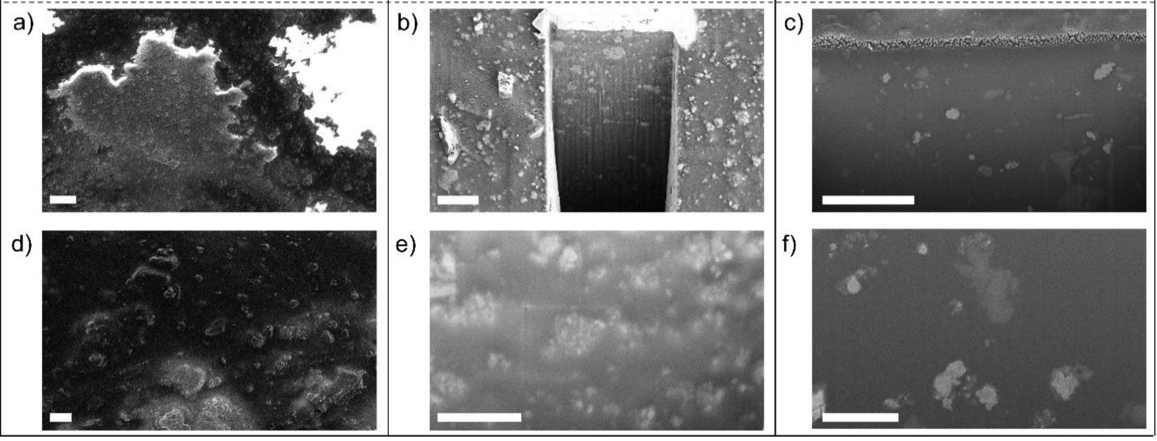

Particle Size Distribution of MOF Dispersions

To determine the best suited conditions for mechanical dispersion, different solvents (tetrahydrofuran (THF) and ethanol (EtOH)), times (1 h, 1.5 h and 3 h) and frequencies (10 Hz and 15 Hz) were investigated with CPO-27-Ni. The resulting PSD for all conditions is narrow and unimodal (centered at ~1.5 μm, **Table S1** and **Figure S3**), but overall, dispersion in ethanol, sonication of the mixture for 3 min and ball milling at 15 Hz for 3 h were selected as the best conditions to obtain the narrowest PSD centered at ~1.22 μm for CPO-27-Ni. Applying the optimized conditions to CuBTTri results in a PSD centered at ~3.99 μm (**Table S1** and **Figure S4**), which is likely due to the large single octahedral crystallite morphology occurring in CuBTTri.

Table S2: Particle size measurements for CPO-27-Ni powder. Shown are values for mechanically dispersed materials with and without sonication. Data is averaged over 3 measurements, where d(0.1) indicates that 10% of the particles are smaller than the given value with 90% being of larger size, d(0.5) is representative of the average size of the particles in the sample and d(0.9) indicates that 90% of the particles are smaller than the given value with 10% being of larger size.

**MOF powder**	**Preparation**	**Particle Size / μm**		
		**d(0.1)**	**d(0.5)**	**d(0.9)**
CPO-27-Ni	dispersed in THF, 1h@15Hz	0.69	1.68	4.74
CPO-27-Ni	dispersed in THF, sonicated 3 min, 1h@15Hz	0.70	1.58	3.79
CPO-27-Ni	dispersed in THF, 1h@10Hz	0.66	1.62	4.57
CPO-27-Ni	dispersed in THF, sonicated 3 min, 1h@10Hz	0.69	1.55	3.51
CPO-27-Ni	dispersed in EtOH, 1.5h@15Hz	0.70	1.49	3.79
CPO-27-Ni	dispersed in EtOH, sonicated 3 min, 1.5h@15Hz	0.68	1.40	3.14
CPO-27-Ni	dispersed in EtOH, 3h@15Hz	0.62	1.30	2.92
CPO-27-Ni	dispersed in EtOH, sonicated 3 min, 3h@15Hz	0.66	1.22	2.28
CuBTTri	dispersed in EtOH, 3h@15Hz	1.31	4.96	17.49
CuBTTri	dispersed in EtOH, sonicated 3 min, 3h@15Hz	1.09	3.99	17.76

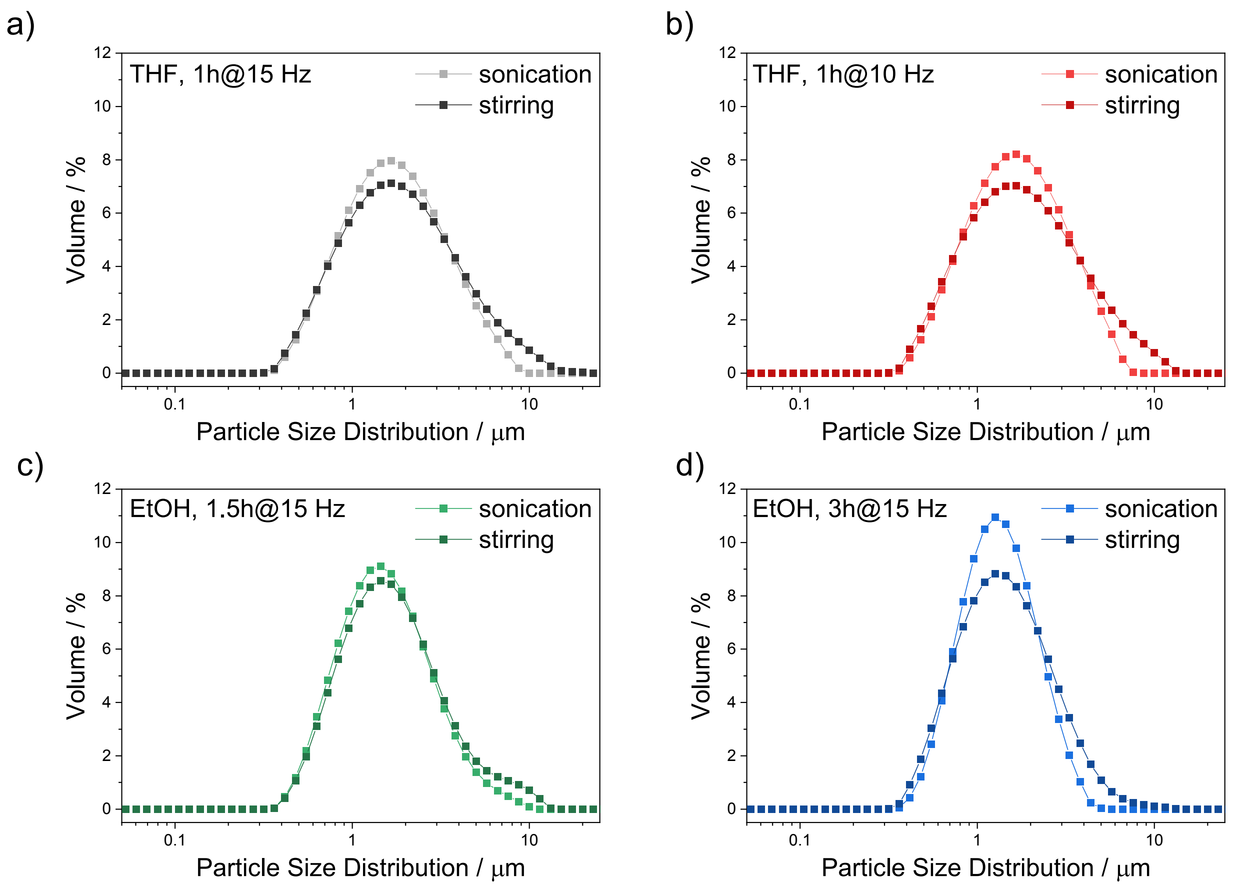

Figure S2: Particle size distribution of CPO-27-Ni slurries after mechanical dispersion in a) tetrahydrofuran (THF) for 1h@15Hz (dark gray – under stirring; light gray – after 3 min sonication); b) THF for 1h@10Hz (dark red – under stirring; light red – after 3 min sonication); c) ethanol (EtOH) for 1.5h@15Hz (dark green – under stirring; light green – after 3 min sonication), and d) ethanol for 3h@15Hz (dark blue – under stirring; light blue – after 3 min sonication), aliquots of the slurry were dispersed in water for measurement. Each curve is an average of 3 measurements.

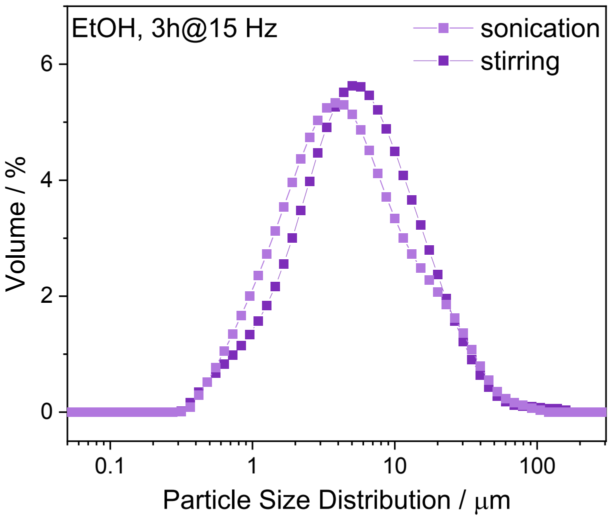

Figure S3: Particle size distribution of CuBTTri slurries after mechanical dispersion in ethanol (EtOH) for 3h@15Hz (dark purple – under stirring; light purple – after 3 min sonication), aliquots of the slurry were dispersed in water for measurement. Each curve is an average of 3 measurements.

2. NO-Release Studies

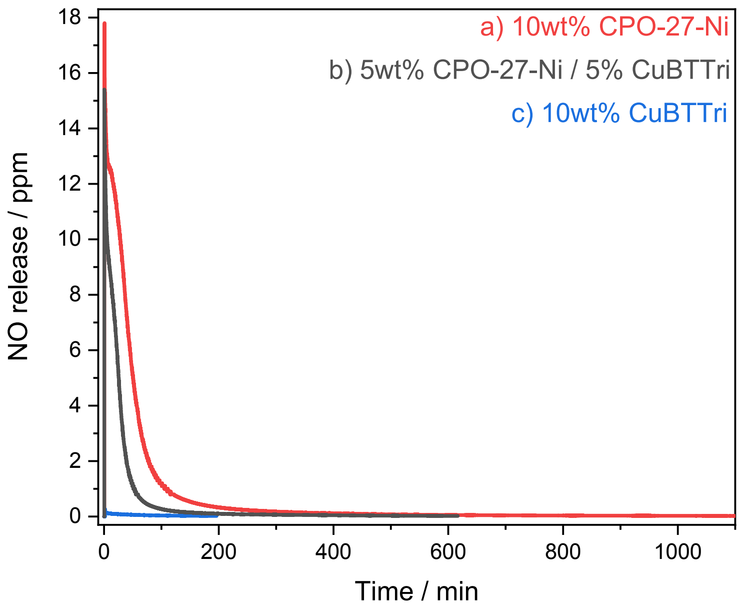

Figure S4: NO release profiles expressed as ppm against time for the MOF composites with a CPO-27-Ni to CuBTTri ratio of a) 10wt%:0wt% (red), b) 5wt%:5wt% (grey) and c) 0wt%:10wt% (blue).

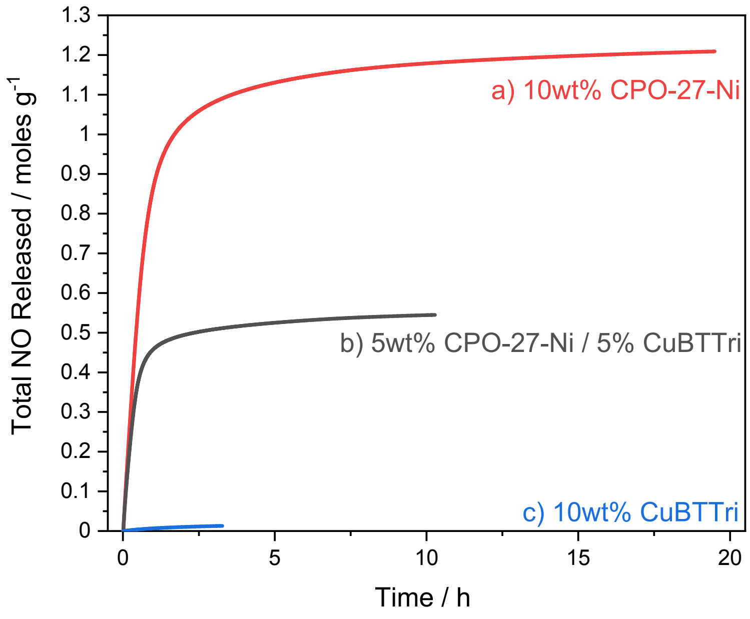

Figure S5: Kinetic NO release profiles expressed as total number of NO released per gram of MOF composites with a CPO-27-Ni to CuBTTri ratio of a) 10wt%:0wt% (red), b) 5wt%:5wt% (grey) and c) 0wt%:10wt% (blue).

Table S3: Comparison of the NO release from the different MOF composites obtained either kinetically by CPO-27-Ni or generated catalytically by CuBTTri.

Ratio CPO-27-Ni: CuBTTri	Kinetic NO release / h	Kinetic NO release / mmol g^{-1}	Catalytic generation of NO
**10wt%:0wt%**	**19.3**	**1.20**	**n/a**
**5wt%:5wt%**	**9.8**	**0.54**	✓
**0wt%:10wt%**	**3.2**	**0.01**	✓
**Blank PU**	**1**	**1.1·10^{-5}**	**–**

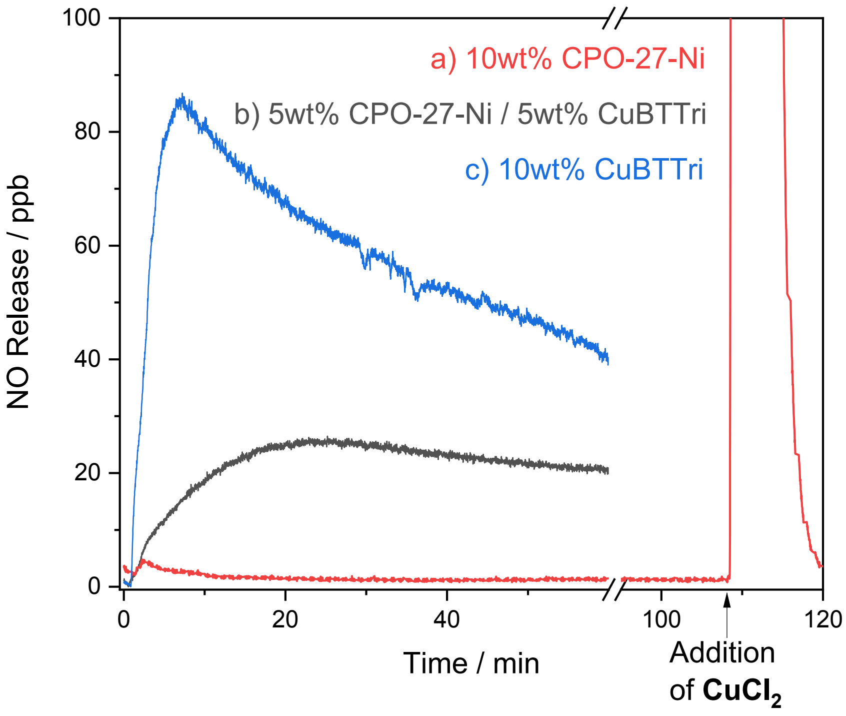

Figure S6: NO release profiles expressed as ppb against time for the MOF composites with a CPO-27-Ni to CuBTTri ratio of a) 10wt%:0wt% (red), b) 5wt%:5wt% (grey) and c) 0wt%:10wt% (blue) used in GSNO to NO catalysis experiments.
